# Do residents in cardiology need more training to make them talk about sex?

**DOI:** 10.1007/s12471-014-0517-0

**Published:** 2014-01-31

**Authors:** M. P. J. Nicolai, H. W. Elzevier

**Affiliations:** 1 Department of Surgery, Westfries Gasthuis, PO Box 600, 1620 AR Hoorn, the Netherlands; 2 Department of Urology, Leiden University Medical Center, Leiden, the Netherlands

**Keywords:** Residents training, Erectile dysfunction, Cardiovascular disease, Quality of care

The editorial comment of last December’s issue of the Netherlands Heart Journal pointed to our paper in that same issue, in which Dutch cardiologists’ knowledge about the effects of cardiovascular agents on sexual function was evaluated (1, 2).

Our previous work had already shown that 42 % of cardiologists indicated a need for training to increase their knowledge about sexual function in patients with cardiovascular disease (3). Concerning that finding, the editors posed the relevant question whether it was mostly the residents indicating this need for training, or not. If that were the case, this implicates that amendments in residents’ training have to be made.

In order to answer this question, we have re-analysed our data and present the results here.

Of all NVVC members, 414 questionnaires were eligible for analysis: 19.1 % were residents in cardiology and 80.9 % were cardiologists. The residents said they discuss sexual function significantly less often with their patients than the cardiologists (linear-by-linear association *p* < 0.001) and referred a significantly lower percentage of patients for treatment of sexual dysfunction (estimated mean 0.9 % ±1.74 compared with cardiologists mean 2.3 % ±4.56; *p* = 0.007). Residents counselled patients with heart failure or after myocardial infarction less often about safely restoring sexual activity (linear-by-linear association *p* = 0.024) or about the use of nitrates when anginal pain occurs during sexual activity (linear-by-linear association *p* = 0.013). Furthermore, residents spoke significantly less often about PDE-5 inhibitor use compared with their bosses (linear-by-linear association *p* < 0.001).

The knowledge about sexual health that residents reported to possess was comparable with that of cardiologists: 71.8 % of the residents said they have sufficient knowledge to be able to discuss sexual function with patients, versus 70.4 % of the cardiologists. The other 28.2 % of residents said they lacked this knowledge (compared with 29.6 % of cardiologists; *p* = 0.80).

Interestingly, only 21.8 % of the residents stated a need for training to be able to provide care for sexual health, compared with 78.2 % of the cardiologists (*p* = 0.23). From the above results, we can infer that Dutch cardiology residents are not motivated to inform patients about sexual function or to provide treatment options for sexual dysfunction.

Yet, our latest study among male patients from four Dutch cardiac clinics indicated that erectile dysfunction (ED) was prevalent in the majority (70 %) of the men with cardiovascular disease; it was the most stated reason not to be sexually active. ED caused significant burden in those affected by it, and 46 % would like to talk about treatment options with the cardiologist. Furthermore, 55 % said it would be helpful if questions about sexual health could be asked during consultation with a specialised nurse and 58 % would appreciate written information (4).

These data, in addition to the mounting evidence published in the last decade (5–7), have shown that ED is a very common problem among men with cardiovascular disease. In addition, it might be aggravated by insecurity about the cardiac condition or induced by the antihypertensive agents used (8). And, although still understudied, sexual dysfunction has shown to be very prevalent among women as well (9, 10). It is therefore impossible for cardiologists to avoid paying attention to sexual health. More training is mandatory to make sure cardiologists are able to inquire about sexual function, to counsel patients about sexual activity after cardiac events, to have the necessary knowledge about medication-induced sexual dysfunction and to prescribe PDE-5 inhibitors. This sub-analysis among Dutch residents in cardiology emphasised that care for sexual health should especially get more attention in residents training.

## References

[1] De Vries Feyens CA, Cramer MJ (2013). Let’s talk about sex. Neth Heart J.

[2] Nicolai MP, Liem SS, Both S (2013). What do cardiologists know about the effects of cardiovascular agents on sexual function? A survey among Dutch cardiologists. Part I. Neth Heart J.

[3] Nicolai MP, Both S, Liem SS (2013). Discussing sexual function in the cardiology practice. Clin Res Cardiol.

[4] Nicolai MPJ, Bavel J, Somsen GA, et al. Erectile dysfunction in the cardiology practice: a patient’s perspective. Am Heart J. 2014; Ref Type: Generic. 10.1016/j.ahj.2013.10.021 24439978

[5] Baumhakel M, Schlimmer N, Kratz M (2011). Cardiovascular risk, drugs and erectile function–a systematic analysis. Int J Clin Pract.

[6] Levine GN, Steinke EE, Bakaeen FG (2012). Sexual Activity and Cardiovascular Disease: A Scientific Statement From the American Heart Association. Circulation.

[7] Nehra A, Jackson G, Miner M (2012). The Princeton III Consensus recommendations for the management of erectile dysfunction and cardiovascular disease. Mayo Clin Proc.

[8] Nicolai MP, Liem SS, Both S (2014). A review of the positive and negative effects of cardiovascular drugs on sexual function: a proposed table for use in clinical practice. Neth Heart J.

[9] Addis IB, Ireland CC, Vittinghoff E (2005). Sexual activity and function in postmenopausal women with heart disease. Obstet Gynecol.

[10] Doumas M, Tsiodras S, Tsakiris A (2006). Female sexual dysfunction in essential hypertension: a common problem being uncovered. J Hypertens.

